# *Ide* copy number variant does not influence stroke severity in 2 C57BL/6J mouse models nor in humans. An exploratory study

**DOI:** 10.1161/STROKEAHA.124.049575

**Published:** 2025-01-27

**Authors:** Marco Foddis, Sonja Blumenau, Susanne Mueller, Clemens Messerschmidt, Clarissa Rocca, Alistair T Pagnamenta, Katarzyna Winek, Matthias Endres, Andreas Meisel, Arianna Tucci, Jose Bras, Rita Guerreiro, Dieter Beule, Ulrich Dirnagl, Celeste Sassi

**Affiliations:** 1 Department of Experimental Neurology, Center for Stroke Research Berlin (CSB), Charité - Universitätsmedizin Berlin, Corporate Member of Freie Universität Berlin, Humboldt-Universität zu Berlin, and Berlin Institute of Health, Berlin, Germany; 2 Berlin Institute of Health, BIH, Core Unit Bioinformatics, Berlin, Germany; 3 Department of Neuromuscular Disease, Queen Square Institute of Neurology, University College London, London, United Kingdom; 4 Wellcome Centre for Human Genetics, Oxford University, Oxford, Oxfordshire, UK; 5 William Harvey Research Institute, Queen Mary University of London, London, United Kingdom; 6 Department of Neurodegenerative Science, Van Andel Institute, Grand Rapids, MI 49503, USA; 7 Charité-Universitätsmedizin Berlin, NeuroCure Cluster of Excellence and Charité Core Facility 7T Experimental MRIs, Berlin, Germany

**Keywords:** *Ide* (insulin degrading enzyme), copy number variant, C57BL/6J mouse strain, bilateral common carotid artery stenosis (BCCAS), middle cerebral artery occlusion (MCAO), posterior communication artery (PcomA) patency, next generation sequencing

## Abstract

**Background:**

Contrary to the common belief, the most commonly used laboratory C57BL/6J mouse inbred strain presents a distinctive genetic and phenotypic variability and for several traits the genotype-phenotype link remains still unknown. Recently, we characterized the most important stroke survival factor such as brain collateral plasticity in two brain ischemia C57BL/6J mouse models (bilateral common carotid artery stenosis and middle cerebral artery occlusion) and observed a Mendelian-like fashion of inheritance of the posterior communicating artery (PcomA) patency. Interestingly, a copy number variant (CNV) spanning *Ide* locus was reported to segregate in an analogous Mendelian-like pattern in the C57BL/6J colonies of the Jackson Laboratory. Given *IDE* critical role in vascular plasticity, we hypothesized *Ide* CNV may have explained PcomA variability in C57BL/6J inbred mice.

**Methods:**

We applied a combination of techniques (T2-weighted magnetic resonance imaging [MRI], time of flight [TOF] angiography [MRA], cerebral blood flow [CBF] imaging and histology) to characterize the collaterome in 77 C57BL/6J BCCAS, MCAO, naïve and sham mice and performed on these Taqman genotyping, exome sequencing, and RNA sequencing. We then investigated the hypothesis that *IDE* structural variants (CNVs, gain/loss of function mutations) may have influenced the cerebrovascular phenotype in a large cohort of 454,040 cases and controls (UK Biobank, Genomics England).

**Results:**

We detected an *Ide* CNV in a BCCAS mouse with 2 patent PcomAs (MAF 1.3%), not segregating with the PcomA patency phenotype. Additionally, two heterozygous *IDE* CNVs, resulting in LoF were found in one patient with hereditary ataxia, a patient with hereditary congenital heart disease and two healthy individuals (MAF 9×10^-6^). Moreover, we report four *IDE* LoF point mutations (p.Leu5X, p.Met394ValfsX29, p.Pro14SerfsX26, p.Leu889X, MAF 0.02%) present also in controls or inherited from healthy parents.

**Conclusion:**

*Ide* CNV and LoF variants are rare, do not crucially influence PcomA variability in C57BL/6J inbred mice and do not cause a vascular phenotype in humans.

## Graphical abstract

**Graphic abstract:**
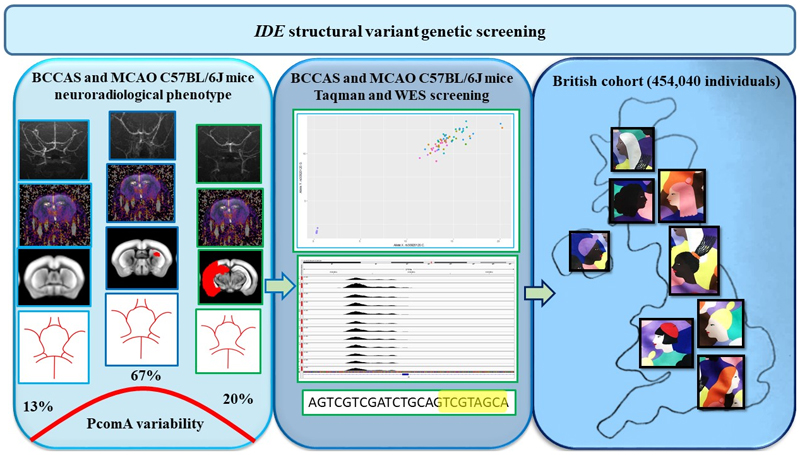

## Nonstandard Abbreviations and Acronyms

AD
: Alzheimer’s disease
BCCAS
: bilateral common carotid artery stenosis
CBF
: cerebral blood Flow
CNV
: copy number variant
IDE
: insulin degrading enzyme
LoF
: loss of function
MCAO
: middle cerebral artery occlusion
MRI
: magnetic resonance imaging
PcomA
: posterior communicating artery

## Introduction

Despite seven decades of inbreeding through several hundreds of brother-sister mating generations, inbred mice, widely used as experimental model of disease, remain only virtually and utopically isogenic ^1, 2, 3, 4, 5^. In the past ten years deep genotyping and next generation sequencing triggered a turbulent wave of genetic discoveries and unveiled a wide spectrum of genetic variants: from synonymous non-coding variants to kilo-megabase copy number variants ^1, 5^ and the meticulous observation of researchers pointed to a variegate intra-and inter-strain phenotypic diversity. This demonstrates that inbred mice, contrary to the common belief, should be considered as members of the same extended multigenerational family, rather than homozygotic twins. In support of these genetic studies, a colourful array of phenotypic traits has been described: from macroscopic differences such as the hair colour, body size, density of the bone mass, to metabolic, behavioural and functional phenotypes, that can only be observed under specific circumstances ^6, 3, 7, 2^. Thus, implying that during decades traits have been positively selected and evolutionary forces gave rise to diverse substrains. Although critical genetic factors have been already found for few of these endophenotypes ^3, 8^, for several of these the genotype-phenotype correlation is yet to be discovered. Among these, we recently reported the variability of the posterior communicating artery (PcomA) patency during acute and subacute brain hypoperfusion in 2 brain ischemia mouse models (bilateral common carotid artery stenosis [BCCAS] and middle cerebral artery occlusion [MCAO]) ^9^. Remarkably, the PcomA recruitment is a dynamic process, represents the most important and main variable survival mechanism and the main determinant of stroke lesion volume and recovery in both models and, in line with previous studies, segregated within the C57BL/6J strain in a Mendelian-like fashion (67% of the mice displayed 1 prominent PcomA, 20% no PcomA and 13% presented 2 very prominent PcomAs) ^9, 10^. Interestingly, among the genetic differences reported in the C57BL/6J strain colonies from the Jackson Laboratory (USA), a ∼ 112 Kb CNV on chromosome 19, encompassing the insulin degrading enzyme gene (*Ide*), associated to a significantly increased *Ide* expression has been analogously reported to be inherited within the C57BL/6J strain in a Mendelian-like pattern (64% of mice heterozygous for the CNV, 23% without CNV and 13% homozygous for the CNV) ^1^. Considering that CNVs represent the main mechanism of genome evolution ^11,12^, given the growing body of evidence pointing to the critical role of genetic structural variants as cause of both common and rare neurological disorders ^13^, *Ide* expression in brain vessel endothelial cells ^14^, *Ide* CNV absence in Balb mice ^15, 1^, which are characterized by a poor collateralization during acute brain hypoperfusion compared to the C57BL/6J strain^16, 17^ and the overlapping pattern of segregation of *Ide* CNV and PcomA patency ^9, 10^ within C57BL/6J strain, we hypothesize that CNVs in *Ide* may explain the diversity of PcomA calibre in the same strain.

**Figure 1:**
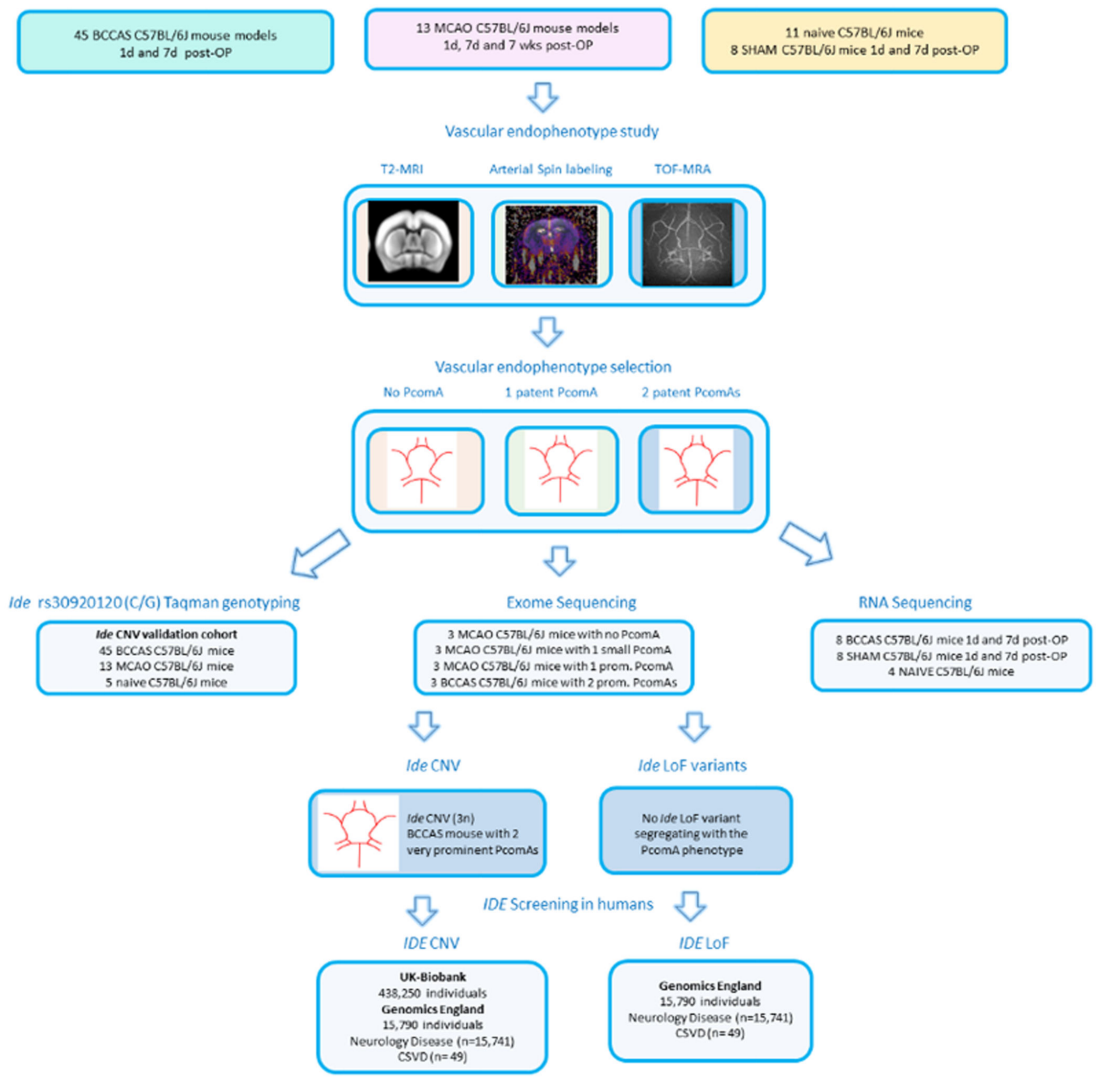
Pipeline followed in our study. We used 2 acute brain ischemia mouse models (BCCAS [bilateral common carotid artery stenosis] and MCAO [middle cerebral artery occlusion]) and studied the posterior communicating artery phenotype (PcomA) using T2 weighted MRI, cerebral blood flow measurement through arterial spin labeling and time-of-flight-angiography (TOF MRA). We then selected BCCAS and MCAO mice with different PcomA phenotype for *Ide* rs30920120 Taqman genotyping, exome sequencing and RNA sequencing. We identified one BCCAS mouse with 2 prominent PcomAs carrying a *Ide* CNV. We screened also the *IDE* gain and loss of function mutations in a human cohort of cases and controls from 2 databases (UK-Brain Bank and Genomics England), where *IDE* CNV and loss of function mutations are very rare and not likely to play a critical influence on vascular phenotypes. D indicates day/s; wks, weeks; CSVD, cerebral small vessel disease; OP, surgery.

To investigate this hypothesis, we applied a combination of complementary techniques (T2-weighted magnetic resonance imaging [MRI], time of flight [TOF] angiography [MRA], cerebral blood flow [CBF] imaging and histology) to characterize brain arterial collaterals in 77 C57BL/6J BCCAS and MCAO mice from Charles River (Germany) and Janvier Laboratories (France), respectively, and genetically characterized these performing Taqman genotyping, exome sequencing and RNA sequencing (Figure 1). We then investigated *IDE* gain and LoF in a large cohort of neurological patients and controls (454,040 individuals from the UK Biobank [438,250 cases and controls] and Genomics England [15,790 neurological patients]).

Importantly, linking the brain arterial collateral plasticity to specific genetic variants in C57BL/6J inbred mice has the enormous potential to provide a unique window into the significantly more complex genetic-phenotypic variability of humans and to effectively enable the detection of robust therapeutic targets.

## Materials and Methods

### Data availability

All data generated or analysed during this study are included in this published article and its supplementary.

### Animals, experimental design and exclusion criteria

Experiments were approved by the Landesamt für Gesundheit und Soziales and conducted according to the German Animal Welfare Act and ARRIVE guidelines (Animal Research: Reporting of In Vivo Experiments, https://www.nc3rs.org.uk/arrive-guidelines). 64 and 15 male C57/BL6 J mice (purchased at 8 weeks of age, Charles River, Germany and 10 weeks of age Janvier France, respectively) were housed in a temperature (22±2°C), humidity (55±10%), and light (12/12-hour light/dark cycle) controlled environment. The animals are subject to brain hypoperfusion between 9 and 13 weeks of age (n=60, BCCAS= 45, MCAO= 15) or were used as controls (naïve=11; BCCAS sham= 8) as previously described ^9^.

The only exclusion criterion was death during MRI caused by the incorrect placement of the animal in the scanner with consequential exclusion of 2 MCAO mice, resulting in final analyzed sample size of MCAO = 13. BCCAS mice were imaged before surgery, 24 hours and 1 week post-surgery. MCAO mice were imaged 24 hours, 1 week, 4 weeks and 7 weeks post-surgery for angiography and estimation of cerebral blood flow (CBF) using arterial spin labeling. At 2 days and 7 days (BCCAS) and 7 weeks (MCAO) tissue was processed for immunohistochemistry

### RNA Isolation for mouse samples and Real-Time PCR

Total RNA from C57BL/6J BCCAS and MCAO blood was isolated using QIAamp RNA Blood Mini Kit (QIAGEN). The quality and the concentration of the total RNA was determined using a Nanodrop Spectrometer (A260:A280 and A260:A230 ratios). For real-time PCR analysis, 750ng total RNA from each sample was used for first-strand cDNA synthesis using SuperScript III (Invitrogen). cDNA from each sample was amplified via real-time PCR and normalized against Actin, using LightCycler 480 Instrument II (Roche). mRNA levels for each experimental group were quantified using the comparative CT method.

### RNA sequencing

Eight BCCAS, 8 sham and 4 näive mice were sacrificed with cervical dislocation 2 days and 7 days post coil insertion surgery, followed immediately by postmortem dissection of the prefrontal cortex, striatum and hippocampus from one hemisphere. The other hemisphere was preserved for immunohistochemistry. The dissected tissues were immersed in RNA later and stored at -80 °C for later use for mRNA-Sequencing. Total RNA was extracted using miRNeasy Kit (Qiagen, Cat # 217004). Total RNA quality was assessed with the use of Bioanalyzer. Average RIN (RNA Integrity Number) of our samples was 9. Next Generation Sequencing mRNA libraries were prepped with Illumina TruSeq RNA Library Preparation Kit (Illumina, Cat # RS-122-2001).

### Mouse cohort for exome sequencing

Our mouse cohort was composed of 9 MCAO mice and 3 BCCAS mice with different collateral circulation phenotype (Table 1, Figure 1).

The study of the PcomA role during acute hypoperfusion followed Martin et al. PcomA classification ^18, 19^ and has been already described ^9^. Briefly, this identifies 4 PcomA classes, based on the ratio between PcomA and basilar artery (BA) diameter: 1) PcomA <10% of BA; 2) PcomA 11-20% of BA; 3) PcomA 21-30% of BA and 4) PcomA >30% of BA. We identify class 1 and class 2 as ’non-patent’, class 3 as ’small ’, class 4 as ’prominent’ and included a fifth class, represented by PcomA>60% of BA, described as ‘very prominent’.

The diameters of the PcomAs were measured at the smallest point and the diameter of the BA was measured proximal to the superior cerebellar arteries both for the Evans Blue and fluorescent WGA stainings (MCAO mice) or only for Evans Blue staining (BCCAS mice) with ImageJ. The diameter of the PcomAs as a percentage of the diameter of the BA was calculated and used in the analysis as previously described ^18, 19^.

In our mouse cohort, 3 BCCAS mice with 2 very prominent PcomAs, together with 9 MCAO mice, characterized by different left PcomA calibre: a) 3 MCAO mice with prominent-very prominent left PcomA that displayed small ischemic lesions (≈5-10% of the left hemisphere), affecting ventral areas (prefrontal cortex, striatum and ventral hippocampus), and presented the most favourable stroke outcomes (Table 1); b) 3 MCAO mice with non-patent PcomA, which survived < 24h post MCAO surgery and c) 3 MCAO mice with small PcomA, that developed monolateral large left strokes affecting up to one third of the left hemisphere and affecting also dorsal areas (orbital cortex and cerebellum) (Table 1) were selected for our exome sequencing study.

Given the extreme inbreeding of the C57BL/6J strain, carefully inbred for over seventy years through more than 200 generations of brother-sister mating ^1^, and the likely minimal influence of environmental factors, these mice were genetically considered as members of the same large multigenerational family coming from a small and isolated village. Moreover, the selection of extreme phenotypes (absent-small PcomA vs prominent-very prominent PcomA), allowed us to reach an high power for the detection of rare variants with large effect size, despite the small sample size ^20, 21^, although no formal sample size/power calculation was performed due to the exploratory nature of the study.

### Exome sequencing in C57BL/6J mice

We performed whole exome sequencing (WES) on a cohort of 12 C57BL/6J mice (9 MCAO and 3 BCCAS). DNA was extracted from cerebellum using standard protocols. Library preparation for next generation sequencing used 50 ng DNA. Exome libraries were prepared using Nextera® Rapid Capture Exome and Kit (4 rxn × 12 plex, FC-140-1002) and Nextera DNA Library Prep Kit (FC-121-1030). The DNA library was then hybridized to an exome capture library (Nextera, Illumina Inc.) and precipitated using streptavidin-coated magnetic beads (Nextera, Illumina). Exome-enriched libraries were PCR-amplified, and then DNA hybridized to paired-end flow cells using a cBot (Illumina, Inc.) cluster generation system.

The WES libraries were sequenced paired-end 75 bp on Illumina HiSeq 4000 with a median of 60.5 million reads per library.

### Bioinformatics, RNA sequencing in C57BL/6J mice

Processing, quality assessment and analysis of RNAseq data was carried out using a custom pipeline. We aligned paired end reads with STAR ^22^ against the GRCm38.p4 genome using gencode.vM12 annotation ^23^, excluding alternative scaffolds and patches. Gene counts were determined using HTSeq ^24^. Testing for differential gene expression and cerebral blood flow and gene-expression correlation was done using DESeq2 ^25^. Genes were counted as differentially expressed where they had a moderated fold change of 2 or more, contrasting coil to shame samples and where their false discovery rate (FDR) adjusted p-value was below 0.05.

### Bioinformatics, exome sequencing in C57BL/6J mice

The reads were aligned using BWA-MEM v0.7.15 ^26^ to the reference GRCm38.p4, separate read groups were assigned for all reads from one lane, and duplicates were masked using Samblaster v0.1.24^27^. Standard QC was performed using FastQC (http://www.bioinformatics.babraham.ac.uk/projects/fastqc). The variants were then called using GATK UnifiedGenotyper v3.7^28^ and annotated using Jannovar v0.24^29^ using RefSeq v105 exons.

For the CNV analysis of the WES data, Cnvkit (https://cnvkit.readthedocs.io/en/stable/) in batch mode was used in a matched fashion as described in their manual for WES data.

All methods were carried out in accordance with relevant guidelines and regulations.

### *IDE* genetic screening in a human cohort

#### CNVs overlapping *IDE* in the UK Biobank and in the Genomics England databank

CEL files from 438,250 individuals were downloaded and CNVs were called using both Affymetrix Power Tools and PennCNV. These individuals were not selected based on any phenotype or diagnosis.

CNVs were considered if including at least 10 SNPs and if at least 50kb in length. Adjacent CNVs were merged based on the default parameters, and CNVs were excluded if they were overlapping telomeres, centromeres, known segmental duplications, immunoglobulin, or T cell receptor loci. PennCNV was used to identify genes which overlapped the CNV calls.

CNVs in *IDE* gene in the genomics England database were investigated as previously described ^30^

#### Loss of function variants in the Genomics England database

We analysed data from the 100,000 Genomes Project (*The National Genomic Research Library v5.1, Genomics England*. https://doi.org/10.6084/m9.figshare.4530893.v7) for families where affected individuals harboured loss of function variants in *IDE*. All genomes from probands and affected family members (n=15,790) recruited under the ‘Neurology Disease’ group (n=15,741) and ‘Familial cerebral small vessel disease’ (n= 49) in the 100KGP were annotated (NM_004969.4) and analysed for *IDE* variants. Then we filtered the dataset for loss of function variants with allele frequency below 0.01.

### MRI Data Analysis in C57BL/6J mice

Cerebral blood flow and angiography CBF maps were calculated using the Perfusion ASL macro in Paravision 5.1 software via the T1 method using a blood T1 value of 2100 ms and a brain blood partition coefficient of 0.89 mL/g 1,2. A custom written Matlab Release 2013a (MathWorks, Natick, MA, USA) script extracted the CBF maps from Paravision, and prompted manual delineation of regions of interest (ROI) in the striatum and prefrontal cortex. The resulting CBF values were expressed in mL/min/100g.

Angiography images were analyzed as previously described ^31^. Briefly, the spatial dimensions or the raw data was up-scaled by a factor of ten, exported into FSL software (Analysis Group, FMRIB, Oxford, UK), and the FLIRT tool was used for coregistration. Registered images were exported into ImageJ freeware (National Institutes of Health) and a maximum intensity projection (MIP) of the Circle of Willis was prepared with a custom plugin. A threshold (14 000 in 16 bit images, i.e. ∼43% of max) was used to create a binary image of the MIP so that the number of voxels in the Circle of Willis could be counted and expressed in µm2.

Ventricle to brain ratio (VBR) and hippocampal size were calculated from the T2 weighted images. Outlines of all structures were manually delineated on a slice by slice basis in ImageJ, and total volumes of each were calculated by multiplying each area by slice thickness (0.50 mm) and summation.

Fisher’s exact test on lesion volume and CBF values was performed. A p-value of 0.05 was set as a nominal significance threshold. All computations, were performed in R (version x64 3.0.2, http://www.r-project.org/).

### Taqman genotyping in C57BL/6J mice

*Ide* genotype rs30920120 (C/G) was assayed using LightCycler 480 Instrument II (Roche) or the TaqMan method (Applied Biosystems Inc. [ABI], Foster City, CA,USA). SNP-specific primers and probes were designed by Thermofischer or ABI (TaqMan genotyping assays). TaqMan real-time polymerase chain reaction assays (PCR) consisted in 2.5 ul of Fast Master Mix (Roche), 0.125 ul of assay, 0.375 ul of water and 2ul of DNA at 5ng/ul. The 5μl total volume reaction was loaded in 384-well plates and was performed in a LightCycler 480 Instrument II (Roche), using a cycling program of: 95°C for 10 min; 40 cycles of 95°C for 15 sec and 60°C for 1 min. Six positive controls, one for each genotype, and one negative control (water) were included in each plate and were consistently called correctly.

### Methods to prevent bias

This is an exploratory, descriptive study. Sample sizes were not based on *a priori* power calculation. Mice were randomized to receive hypoperfusion. The study was only partially blinded.

## Results

In this study we tested the hypothesis that the phenotypic correlate of *Ide* duplication and gene expression variability reported in C57BL/6J inbred strain from the Jackson Laboratory ^1^ may have been the different dynamic PcomA patency degree, described in the same strain with overlapping frequency^9^.

To test this hypothesis we used a combination of complementary genetic techniques (Taqman genotyping, exome sequencing and RNA sequencing) in two different C57BL/6J mouse models of brain acute and subacute hypoperfusion: BCCAS and MCAO (Figure 1).

### Taqman genotyping of *Ide* rs30920120 of BCCAS and MCAO and naïve C57BL/6J mice with different PcomA patency features

**Figure 2:**
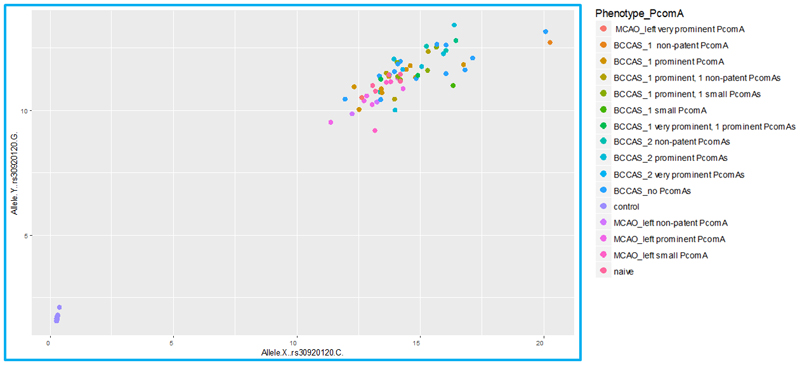
*Ide* rs30920120 (C/G) Taqman genotyping in a cohort of bilateral common carotid artery stenosis (BCCAS), middle cerebral artery occlusion (MCAO) and naïve C57BL/6J mice with different posterior communicating artery (PcomA) phenotype.

We selected the *Ide* rs30920120 probe, corresponding to the coding SNP C/G on chromosome 19 at *Ide* locus, and described by Watkins-Chow and colleagues as not showing identical heterozygosity in the C57BL/6J mouse strain instead falling in 2 discrete genotype classes differing in their signal intensity ratio ^1^. We performed Taqman genotyping using the probe rs30920120 on a cohort of 63 C57BL/6J mice: 45 BCCAS mice (71%) 2 days or 7 days post surgery, 13 MCAO mice (21 %) 1 day, 7 days or 7 weeks post-surgery and 5 naïve mice (8%), which did not display any patent PcomA (Table 1). All the samples displayed identical heterozygosity and overlapping signal intensity, without falling into two discrete genotype classes, as described in Jackson laboratory C57BL/6J mice ^1^(Figure 2).

We then performed whole exome sequencing in 12 C57BL/6J MCAO and BCCAS mice that showed phenotypically significantly different stroke lesion sizes, collateral blood flow and arterial brain collateral recruitment pattern (Figure 1)

### *Ide* CNV detection in exomes of BCCAS and MCAO mice with different PcomA phenotype

We performed exome sequencing in 9 MCAO and 3 BCCAS mice with diverse PcomA caliber and investigated the hypothesis that PcomA spectrum size, ranging from no PcomA/non-patent PcomA to very prominent PcomAs may have been determined by CNVs or LoF in *Ide* (Figure 1, Table 1).

**Figure 3:**
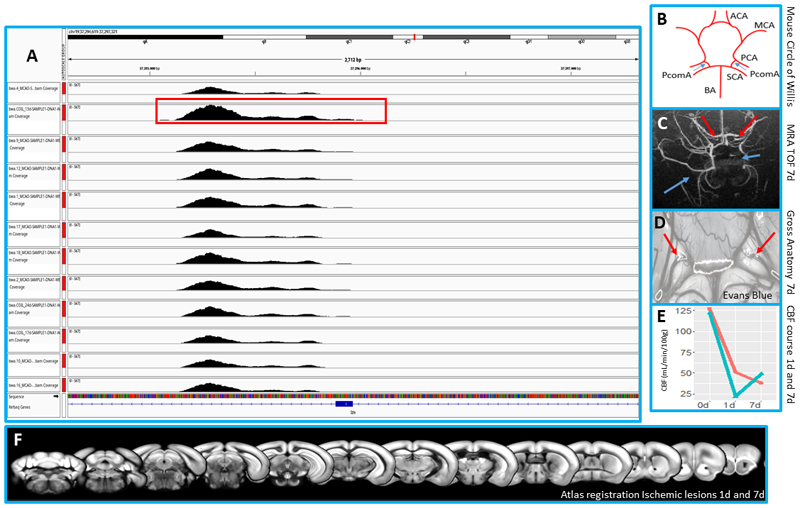
*Ide* copy number variant (CNV) detection in the C57BL/6J BCCAS and MCAO exome sequencing mouse cohort and *Ide* CNV carrier phenotype. **A**. CNV analysis of *Ide* locus, based on exome sequencing data on 12 BCCAS and MCAO mice with different PcomA phenotype. In the red rectangle is underlined the *Ide* CNV detected in a BCCAS mouse. **B-F**. Neuroradiological phenotype of the BCCAS mouse carrying *Ide* CNV. **B** Schematic representation of the circle of Willis, displaying a complete circle with 2 prominent PcomAs (blue arrows). ACA, anterior communicating artery; MCA, middle cerebral artery; PCA, posterior cerebral artery; SCA, superior cerebellar artery; BA, basilar artery; PcomA, posterior communicating artery. **C**. Time of Flight (TOF) angiography showing 2 patent PcomAs (red arrows) 7 days post surgery and secondary collateralization from the external carotid artery (blue arrows). **D**. Gross anatomy of the BCCAS mouse carrying the *Ide* CNV, 7 days post surgery and after Ivans Blue injection, presenting 2 very prominent PcomAs (red arrows). **E**. Graph showing initial cerebral blood flow drop 24 hours post surgery and progressive recovery 7 days post surgery, through brain collaterals. The blue segment represents the right circulation and the red segment the left circulation measured by arterial spin labelling. D, day; CBF, cerebral blood flow. **F**. Atlas registration overlap at different time points (2 days and 7 days post surgery) of the BCCAS mouse carrying *Ide* CNV, showing no ischemic lesions.

We report an *Ide* CNV (3n) (Figure 3A) in a BCCAS mouse with 2 effective PcomAs (Figure 3B-F), not segregating with the patent-PcomA phenotype.

We next focused on loss of function mutations in *Ide* as another likely genetic mechanism potentially explaining intra-strain vascular differences in our mouse exome cohort and did not detect any *Ide* LoF mutation segregating with the PcomA phenotype.

**Figure 4:**
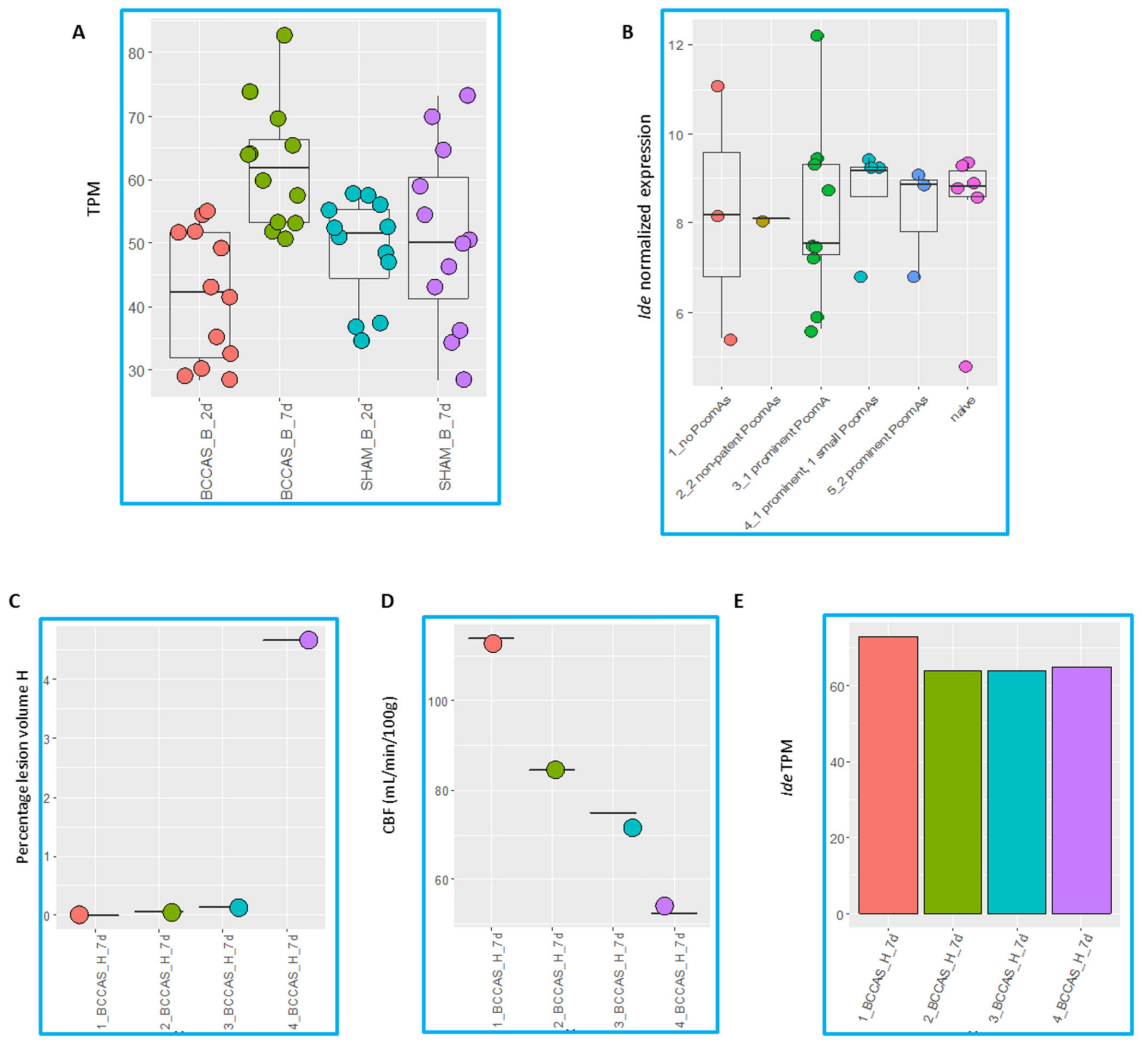
*Ide* gene expression analysis in BCCAS and MCAO mice during acute (2 days) and subacute (7 days) hypoperfusion with different PcomA patency pattern, hippocampal lesion sizes and cerebral blood flow recovery. **A**. RNA sequencing *Ide* brain expression analysis in BCCAS and SHAM mouse brain (B; prefrontal cortex, hippocampus and striatum) during acute and subacute hypoperfusion. **B**. Real-TIME PCR *Ide* expression analysis in BCCAS, MCAO and naïve mice with different PcomA patency in blood during acute hypoperfusion. **C-E**. Hippocampal percentage of lesion volume (**C**), CBF measurements (CBF, ml/min/100g) (**D**) and *Ide* gene expression (RNA sequencing, **E**) in BCCAS mice 7 days post-surgery. H, hippocampus; TPM, transcript per million and d, days.

Finally, since the *Ide* CNV reported in the Jackson Laboratory colonies corresponds to a proportional increase in *Ide* gene expression we further examined firstly, whether during brain hypoperfusion there was a significant increase of *Ide* expression in BCCAS mice in the most hypoperfused brain areas (Figure 4A) and secondly, whether there may have been a correlation between *Ide* differential gene expression and PcomA patency (Figure 4B), ischemic lesion sizes and cerebral blood flow recovery (Figure 4 C-E). Therefore we performed RNA sequencing during acute (2d) and subacute (7d) brain hypoperfusion in hippocampus, striatum and prefrontal cortex of C57BL/6J BCCAS mice with different PcomA patency and stroke lesion sizes (Figure 1).

### *Ide* expression in hippocampus, striatum and prefrontalcortex in BCCAS C57BL/6J mice during acute and subacute brain hypoperfusion

We did not detect a significant *Ide* differential expression during acute and subacute brain hypoperfusion in hippocampus, striatum and prefrontal cortex between BCCAS and SHAM or naïve mice as well as in blood samples of BCCAS, MCAO and naïve mice with different PcomA patency phenotype (|Fold change|<2, FDR p-value>0.05) (Figure 4 A-B). Moreover, we do not report any correlation between *Ide* expression, severity of the stroke lesion size and rapidity of cerebral blood flow recovery (Figure 4 C-E). Thus, implying that brain diverse collateralization is not shaped by *Ide* expression.

### CNVs overlapping *IDE* in the Genomics England database and UK Biobank

We found 2 *IDE* CNVs in the Genomics England database (chr10:92556744-92561449, chr10:92557209-92565408) that were detected in one patient with hereditary ataxia and with syndromic congenital heart disease, respectively, and both of them were also found in two healthy controls (minor allele frequency [MAF] 9×10^-6^), Table 2). All of these *IDE* CNVs were identified in a nonsense-mediated mRNA transcript (NM_001322795), expressed at very low levels in the skin (Gtex data), the tissue where *IDE* is most abundant.

On the contrary, we did not identify any CNV spanning the *IDE* gene in the 438,250 UKBB participants. After QC, a total of 7 individuals were found as potential CNV carriers overlapping *IDE*. To evaluate the authenticity of these calls, B-allele frequency and Log R Ratios were used to plot the CNVs. Plots were then generated covering *IDE* +/-5Mb but there was no evidence that any of these calls were true CNVs, as can be seen in the plots in the supplementary materials (Figure S1 A-GI)

In line with these findings, only 6 small structural variants and 2 large inversions are present in gnomAD (https://gnomad.broadinstitute.org/, n=21,694 alleles).

### *IDE* LoF Variants in Genomics England Database

In the 15.790 neurological patients analysed in the 100,000 Genomes Project (Genomics England) screened for *IDE* rare loss of function variants (MAF < 0.01), we report 44 patients carrying heterozygous LoF variants in *IDE* (Table 2). Among these, 41 patients carried rs533083105 (p.Leu5X) and one patient carried the rs749353444 (p.Met394ValfsX29), both of them detected also in healthy controls. Two other cases inherited the frameshift variant, p.Leu889X and p.Pro14SerfsX26 from the healthy parents, mother and father, respectively.

## Discussion

In this study we tested the hypothesis that *Ide* CNV (rs30920120) reported in C57B6/6J mice in the colonies of Jackson Laboratory and associated to a proportional *Ide* overexpression ^1^ may explain the variability of the PcomA patency that we reported during acute brain hypoperfusion in this mouse strain ^9^.

Surprisingly, all of the 63 BCCAS, MCAO and naïve C57BL/6J inbred mice from Charles River (Germany, 79%) and from Janvier (France, 21%) colonies displayed identical heterozygosity for the rs30920120 variant and did not present any *Ide* CNV (rs30920120) in our Taqman genotyping (Figure 2). This may be due to the relatively small sample size. Alternatively, it may be possible that the *Ide* CNV described in heterozygosity in 64% of C57BL/6J mice from the colonies of Jackson Laboratory ^1^ may not be present in different breeding laboratories. Thus, raising the intriguing hypothesis that beside a inter-and intrastrain genetic diversity there may be an additive inter-provider genetic diversity, significantly increasing the complexity of genotype–phenotype correlations. Remarkably, this is in line with previous studies describing five-exon deletion in *Nnt* which encodes the nicotinamide nucleotide transhydrogenase (NNT) present in C57BL/6 mice supplied by Jackson laboratory but absent in C57BL/6 mice supplied by Taconic or Charles River ^32^ and with a growing body of evidence suggesting that genetic structural variants in humans are potential substrates for natural selection resulting in phenotypic and population-specific differences ^33,13^.

Importantly, historically archived samples from the C57BL/6J colony suggest that *Ide* duplication has rapidly reached a high frequency in the Jackson laboratory colonies since 1994 ^1^. Notably, the Charles River laboratory received the C57BL/6J colonies in 1974 from the National Institutes of Health (NIH, USA) (https://www.criver.com/) and Janvier Laboratory is an independent family-owned business established in 1960 by Roger Janvier in France (https://janvier-labs.com/en/historical-overview/). Therefore, supporting the hypothesis that *Ide* CNV may be an independent event originated in and peculiar of Jackson Laboratory C57BL/6J colonies.

In our exome sequencing C57BL/6J mouse cohort, we found a *Ide* CNV in a BCCAS mouse that during acute brain ischemia developed 2 very prominent PcomAs (Figure 3A-F). However, *Ide* CNV did not segregate with the PcomA-patency phenotype in the same and different C57BL/6J stroke mouse models (MCAO, Figure 3A). Moreover, *Ide* expression did not significantly differ in BCCAS mice with different stroke outcomes and therefore diverse PcomA endophenotypes (absent/small/prominent/very prominent PcomA or 2 prominent PcomAs) (Figure 4A-E).

Finally, to test the hypothesis that *IDE* gain or loss of function may have influenced a possible vascular phenotype in humans, we screened a cohort of 454.040 individuals for *IDE* CNV and 15.790 neurological patients for *IDE* LoF.

Since both *IDE* CNVs and heterozygous *IDE* LoF mutations in our cohort have been detected either in patients not presenting a pathogenic neurovascular phenotype or in healthy controls or have been inherited from healthy parents (Table 2), we exclude that *IDE* structural changes may critically influence a vascular trait in humans.

Thus, finally, implying that the phenotypic correlate of *Ide* CNV reported in C57BL/6J strain remains still unknown.

Insulin degrading enzyme (*IDE*) is a highly evolutionary conserved protease that has been involved in Insulin and amyloid-beta metabolism and consequentially associated to diabetes mellitus, metabolic syndrome, Alzheimer’s disease and amyloid angiopathy ^34,35,36,37^.

*IDE* represents one of the principal proteases responsible for Aβ clearance ^38^. *IDE* activity in the human brain decreases with aging and in the early stages of Alzheimer’s disease (AD) pathology ^39^. Moreover, even modest overexpression of *Ide* in transgenic mice significantly prevents the formation and deposition of amyloid plaques in mice ^40^.

By contrast, partial *Ide* LoF leads to diabetes and impaired Abeta 42 protein degradation in experimental models ^41^.

Additionally, in the last decades several genetic association studies (GWAS) have identified common single nucleotide polymorphisms (SNPs) within *IDE* locus associated particularly with sporadic late-onset AD in *APOE* ε4 carriers in different populations ^42,43,44,45,46,47,48^. Notably, these SNPs are low penetrant, and generally non-coding variants that likely exert a subtle regulatory effect (0.8 < odds ratio [OR] < 1.5) and contribute to the susceptibility for late-onset AD decreasing *IDE* expression ^49,50,51,52,53,36^. However, *IDE* structural variants have not been extensively investigated.

Thus, considering that C57BL/6J mice are widely used as genetic background of at least 50 different AD mouse strains (https://www.alzforum.org/research-models/alzheimers-disease, Table S1), *Ide* duplication reported in the Jackson colonies may deeply influence interpretations of experimental results and conventional drug testing in mice, particularly in the AD field.

Although in this study we did not find any causative correlation between *IDE* CNV (rs30920120) described in the Jackson Laboratory and PcomA variability in Charles River and Janvier C57BL/6J mice, in our exome sequencing screening we identified a BCCAS mouse with 2 prominent PcomAs carrying a *Ide* CNV (Figure 3). Although this *Ide* CNV did not segregate with the PcomA Phenotype (as was not detected in the other mice with patent PcomAs) to test the hypothesis that *IDE* structural variants and LoF mutations may have played a subtle influence (0.8 < odds ratio < 1.5) in determining a cerebrovascular phenotype, we screened both low frequency and rare *IDE* CNVs and LoF mutations (insertions, deletions and stop gain mutations) in a large cohort of patients and controls.

Finally, the brain collateral flow represents the most important survival factor during acute brain ischemia, determining the stroke lesion size and overall outcome and relies on the recruitment of collateral arteries and, particularly in the C57BL/6J mice, on PcomA patency, which, despite the Mendelian-like pattern of segregation within the strain ^9, 10^ and the numerous genetic studies, remains genetically not characterized.

In summary, our study supports a growing body of evidence pointing to significant genetic and phenotypic differences within the most widely used C57BL/6J mouse strain and suggests that this genetic variability may also depend on the main laboratory animal suppliers. Moreover, our results shows that *Ide* CNVs are rare and do not critically influence PcomA phenotype in C57BL/6J BCCAS and MCAO mouse models and stroke in humans.

Considering that C57BL/6J is the most widely used mouse strain in preclinical studies worldwide and that to date there are 29 different mouse providers (https://biotech-careers.org/company-core-activity/animal-models), our findings have enormous implications for the reproducibility and reliability of scientific studies and should foster additional studies in C57BL/6J mice aimed at 1) improving the genetic quality control in inbred laboratory mice; 2) determining *Ide* CNV phenotypic correlate and 3) exploring the genetic determinants of PcomA caliber, which may provide a unique window into genetic determinants of collaterome in humans.

## Supplementary Material

Supplemental Publication Material

## Figures and Tables

**Table 1:** Exome sequencing and *Ide* rs30920120 (C/G) Taqman genotyping cohort description.

Mouse strain	Mouse model	Vascular Phenotype*	Sex	Age	MRI Pattern	N/Tot (%)	Sequencing/Genotyping strategy
C57BL/6J	BCCAS	2 prominent PcomAs	M	10-12wks	No ischemic lesion	3/12(25) 8/63 (13)	WES Taqman
C57BL/6J	BCCAS	No prominent PcomAs	M	10-12wks	1 severe cortical and subcortical ischemic lesion affecting up to 34% of one hemisphere	18/63 (28)	Taqman
C57BL/6J	BCCAS	1 prominent PcomA	M	10-12wks	1 small subcortical ischemic lesion affecting 1–5% of one hemisphere	17/63 (27)	Taqman
C57BL/6J	BCCAS	1 small PcomA	M	10-12wks	Multiple small bihemispheric lesions	2/63 (3)	Taqman
C57BL/6J	MCAO	Left prominent/very prominent PcomA	M	10-12wks	1 small cortical and subcortical ischemic lesion affecting 5–10% of the left hemisphere	3/12(25) 7/63 (11)	WES Taqman
C57BL/6J	MCAO	Left small PcomA	M	10-12wks	1 severe cortical and subcortical ischemic lesion affecting >20% of the left hemisphere.	3/12(25) 4/63 (6)	WES Taqman
C57BL/6J	MCAO	Left non-patent PcomA	M	10-12wks	1 severe cortical and subcortical ischemic lesion affecting >35% of the left hemisphere	3/12(25) 2/63 (3)	WES Taqman
C57BL/6J	NAÏVE	no PcomAs	M	10-12wks	No ischemic lesion	5/63 (8)	Taqman

* PcomA classification is based on the PcomA/Basilar Artery diameter ratio, already described ^9^. BCCAS, bilateral common carotid artery stenosis; MCAO, middle cerebral artery occlusion; PcomA, posterior communicating artery; wks, weeks; WES, whole exome sequencing; N,number.

**Table 2:** *IDE* LoF and *IDE* CNV mutations detected in the 15.790 genomes from the 100,000 Genomes Project (Genomics England).

Position	rsID	Ref/Alt	cDNA	Aa	Gen	gnomAD_AF	ClinVar	GenE (%)	Phenotype
chr10:92574007	rs533083105	AG/A	c.13del	p.Leu5X	Het	0,000958	Not present	41/15,790 (0.2)	Neurology and Neurodevelopmental disorders/Cardiovascular Disorders*
chr10:92507639-92507640	rs749353444	CAT/C	c.1180_1181del	p.Met394ValfsX29	Het	0,00005172	Not present	1/15,790 (0)	Neurology and Neurodevelopmental disorders*
chr10:92573971-92573980	NA	AAGGTGCTGGG/A	c.40_49del	p.Pro14SerfsX26	Het	NA	Uncertain significance	1/15,790 (0)	Neurology and Neurodevelopmental disorders*
chr10:92463828	NA	AT/ A	c.2664del	p.Leu889X	Het	NA	Not present	1/15,790 (0)	Neurology and Neurodevelopmental disorders*
chr10:92556744-92561449	NA	NA	NA	NA	Het	NA	NA	NA	Hereditary ataxia and control
chr10:92557209-92565408	NA	NA	NA	NA	Het	NA	NA	NA	Syndromic congenital heart disease and control

Aa, ammino-acid; chr, chromosome; Ref/Alt, Reference/Alternate; Gen, genotype; Het, heterozygous, NA, not available.

* Also present in healthy controls

## Summary

We hypothesized that *Ide* CNV may influence the PcomA variability in C57BL/6J ischemia mouse models and stroke risk in a large British cohort and conclude that *IDE* CNV is rare and does not cause a cerebrovascular phenotype in mice nor in humans.
