# Supplementary material for: Ide copy number variant does not influence stroke severity in 2 C57BL/6J mouse models nor in humans. An exploratory study
Source: Stroke. Author manuscript; Available in PMC 2025 May 6. (PMC7617642; doi:10.1161/STROKEAHA.124.049575)
Supplement: Supplemental Publication Material [file EMS202781-supplement-Supplemental_Publication_Material.pdf]

# SUPPLEMENTAL MATERIAL

| AD strain name | Background | Plaque development |
|---|---|---|
| 3xTg | C7BL/6;129X1/SvJ;129S1/Sv | Plaques at 6 months |
| 5xFAD (B6SJL) | C57BL/6 x SJL | Plaques at 3 months |
| 5xFAD (C57BL6) | C57BL6 | Plaques at 3 months |
| A7 APP transgenic | C57BL/6J | Plaques at 6 months |
| ADanPP | C57BL/6J | Plaques at 3 months |
| AD-BXD | C57BL/6J X BXD | Plaques at 6 months |
| APP23 | C57BL/6 | Plaques at 6 months |
| APP23 x PS1-R278I | C57BL/6J | Plaques at 6 months |
| APP751SL/PS1 KI | 129SV x C57BL/6 | Plaques at 3 months |
| APP NL-F Knock-in | C57BL/6 | Plaques at 6 months |
| APP NL-G-F Knock-in | C57BL/6 | Plaques at 3 months |
| AppNL-G-F/MAPT double knock-in | C57BL/6J | Plaques at 2 months |
| APPPS1 | C57BL/6J | Plaques at 3 months |
| APP/PS1/rTg21221 | B6.C3 x B6.129 x FVB | Plaques at 9 months |
| AppSAA Knock-in | C57BL/6J | Plaques at 3 months |
| APPsw/0; Pdgfrβ+/- | APPsw mice on C57BL/6; Pdgfrβ+/- mice on 129S1/SvlmJ | Plaques at 9 months |
| APPSwDI x NOS2 Knock-out | C57BL/6J; C57BL/6N | Plaques at 1 year |
| APPSwe (line C3-3) | C3H/HeJ x C57BL/6J; backcrossed to C57BL/6J | Plaques at 18 months |
| APPSwe/PSEN1(A246E) | C57BL/6J x C3H/HeJ | Plaques at 9 months |
| APPSwe/PSEN1dE9 (C3-3 x S-9) | C57BL/6J | Plaques at 6 months |
| APPswe/PSEN1dE9 | C57BL/6J | Plaques at 3 months |
| APPswe/PSEN1dE9 (line 85) | C57BL/6;C3H | Plaques at 6 months |
| APP(V717I) | Originally generated on FVB/N background; available at reMYND as C57BL/6xFVB/N | Plaques at 9 months |
| APP(V717I) x PS1(A246E) | Originally generated on FVB/N background; available at reMYND as C57BL/6xFVB/N | Plaques at 3 months |
| Arc48 (APPSw/Ind/Arc) | C57BL/6 | Plaques at 2 months |
| ARTE10 | Co-injection of transgenes into B6CBF1 oocytes, back-crossed to C57BL/6 | Plaques at 3 months |
| BACE1 cKO (Hu, Yan) X 5xFAD | C57BL/6J | Plaques at 3 months |
| BRI-Aβ42 (BRI2-Aβ42) | B6C3, backcrossed to C57BL/6J | Plaques at 3 months |
| E2FAD | C57BL/6 | Plaques at 3 months |
| E4FAD | C57BL/6 | Plaques at 4 months |
| J20 (PDGF-APPSw,Ind) | C57BL/6 | Plaques at 5 months |
| mThy1-hAPP751 (TASD41) | C57BL/6 x DBA | Plaques at 3 months |
| PDAPP(line109) | C57B6 x DBA2 | Plaques at 6 months |
| PS2APP (PS2(N141I) x APPswe) | C57BL/6, DLB/2, crossed to C57BL/6 | Plaques at 9 months |
| PS/APP | B6/D2/Swe/SJL mixed background | Plaques at 6 months |
| TAS10 (thy1-APPswe) | Transgene injected into C57BL/6 x C3H oocytes, some backcrossing to C57BL/6 | Plaques at 6 months |
| TASTPM (TAS10 x TPM) | TAS10 transgene originally injected into C57BL/6 x C3H oocytes, with some backcrossing to C57BL/6 | Plaques at 6 months |
| TauPS2APP | C57BL/6, DBA/2; backcrossed to C57BL/6 | Plaques at 4 months |
| Tg2576 | B6;SJL Mixed Background | Plaques at 10 months |
| Tg2576/Tau(P301L) (APPSwe-Tau) | C57BL/6, DBA/2, SJL, SW Mixed Background | Plaques at 9 months |
| tg-APPSwe | C57BL/6J | Plaques at 12 months |
| Tg-ArcSwe | C57BL/6J | Plaques at 6 months |
| Tg-SwDI (APP-Swedish,Dutch,Iowa) | C57BL/6 | Plaques at 3 months |
| TREM2-BAC X 5xFAD | TREM2-BAC: FVB/NJ; 5xFAD: C57BL/6 X SJL | Plaques at 6 months |
| TREM2, humanized (common variant) X 5XFAD | C57BL/6 X CBA, back-crossed for at least 4 generations to C57BL/6 | Plaques at 8 months |
| TREM2, humanized (R47H) X 5XFAD | C57BL/6 X CBA, back-crossed for at least 4 generations to C57BL/6 | Plaques at 8 months |
| Trem2 KO (Colonna) x 5XFAD | C57BL/6Trem2 KO (KOMP) x APPPS1 Genetic Background: C57BL/6 | Plaques at 4 months |
| Trem2 R47H KI (Lamb/Landreth) X APPPS1-21 | C57BL6/J | Plaques at 3 months |

**Table S1. Different Alzheimer's disease (AD) C57BL6/J mouse models and respective neuropathological phenotype.**

**Figure S1**

A **Partecipant A** AI

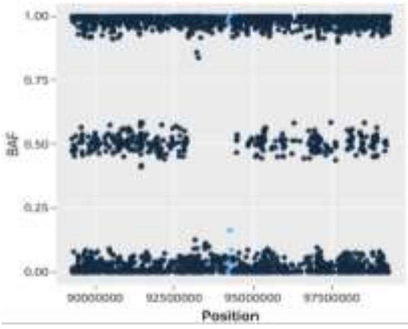


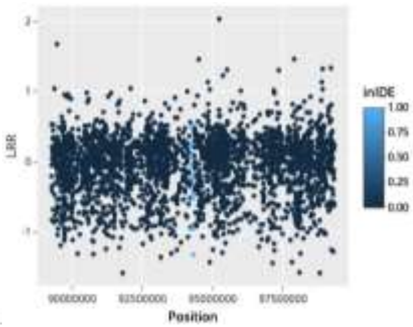


B **Partecipant B** BI

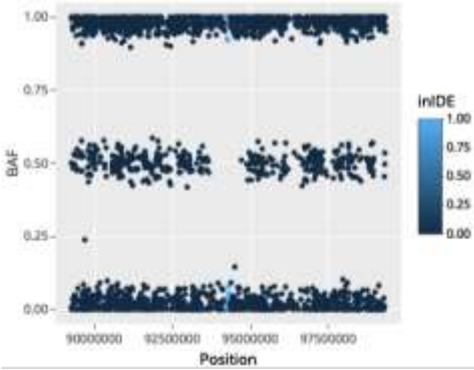


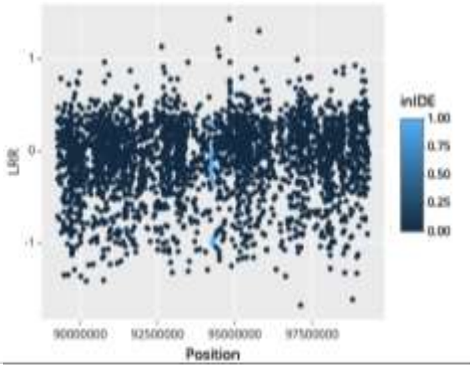


C **Partecipant C** CI

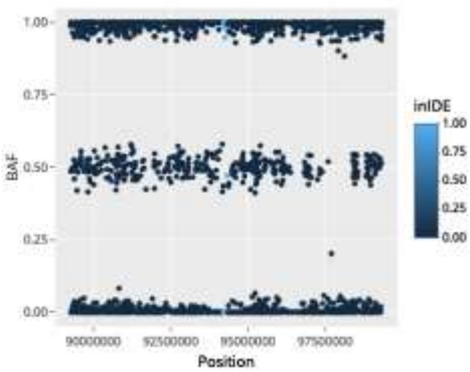


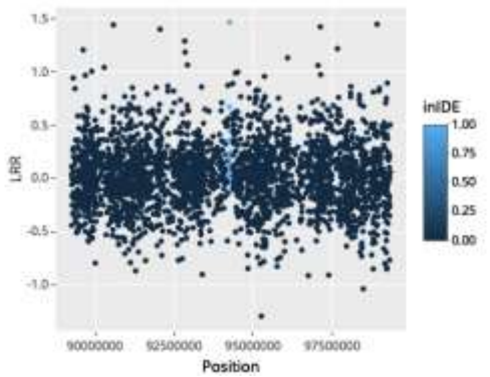


D **Partecipant D** DI

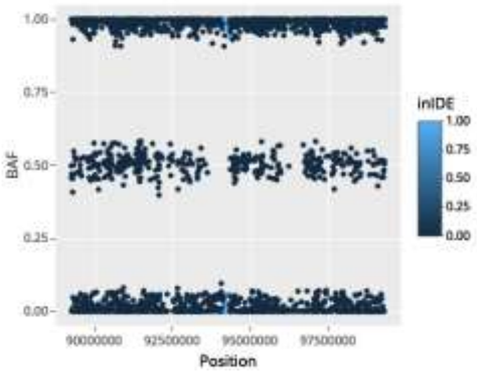


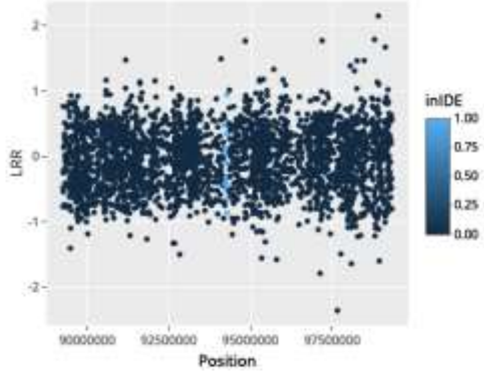

E Partecipant E EI

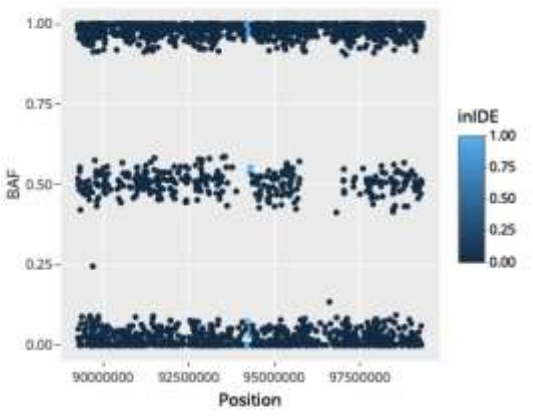


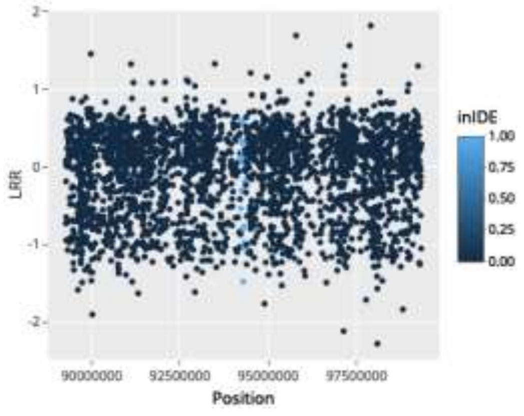


F Partecipant F FI

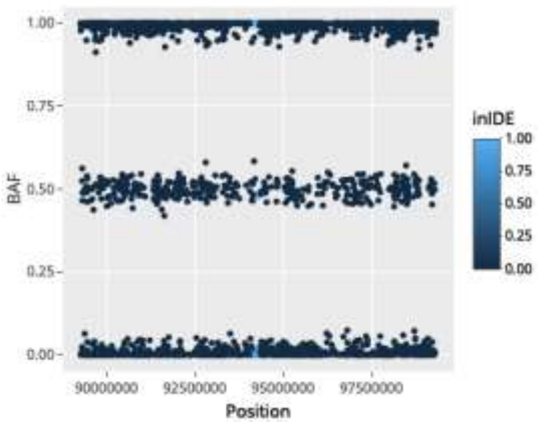


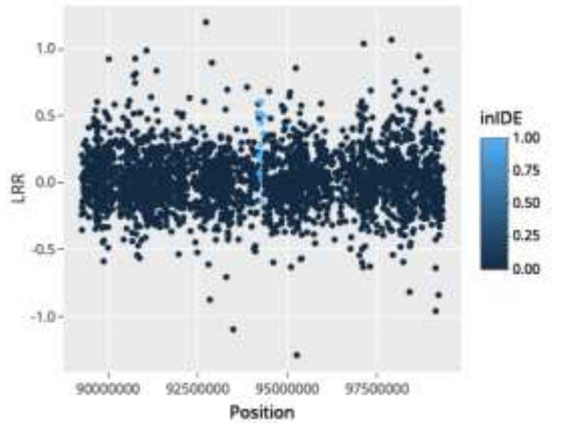


G Partecipant G GI

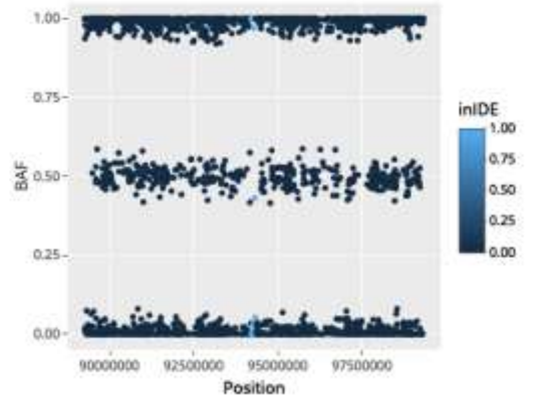


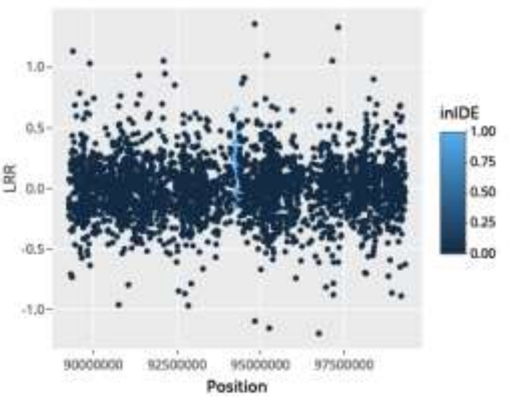


**Figure S1 A-G. Copy number variant (CNV) plots spanning IDE +/-5Mb locus in the 438,250 UKBB participants.** After QC, a total of 7 individuals (participant A-G) were found as potential *IDE* CNV carriers. To evaluate the authenticity of these calls, B-allele frequency (BAF) and Log R Ratios (LRR) were used to plot the CNVs. Plots were then generated covering IDE +/-5Mb but there was no evidence that any of these calls were true CNVs (A-GI)
